# Delta-Like 4 Differentially Regulates Murine CD4^+^ T Cell Expansion via BMI1

**DOI:** 10.1371/journal.pone.0012172

**Published:** 2010-08-17

**Authors:** Matthew A. Schaller, Hannah Logue, Sumanta Mukherjee, Dennis M. Lindell, Ana Lucia Coelho, Pamela Lincoln, William F. Carson, Toshihiro Ito, Karen A. Cavassani, Stephen W. Chensue, Cory M. Hogaboam, Nicholas W. Lukacs, Steven L. Kunkel

**Affiliations:** 1 Department of Pathology, University of Michigan, Ann Arbor, Michigan, United States of America; 2 Center for Immunity and Immunotherapies, Seattle Children's Hospital, Seattle, Washington, United States of America; New York University, United States of America

## Abstract

**Background:**

Studies have shown that Notch is essential for the maintenance of a T cell Th2 phenotype in vivo. It has also been shown that Notch ligands have diverse functions during T cell activation. We chose to investigate the role of Notch ligands during the Th2 response.

**Principal Findings:**

We studied the relationship of two Notch ligands, delta-like 4 and jagged-1, to T cell proliferation in C57 Bl/6 mice. Our findings indicate that jagged-1 does not affect the rate of T cell proliferation in any subset examined. However, delta-like 4 causes an increase in the expansion of Th2 memory cells and a decrease in effector cell proliferation. Our in vivo studies indicate that the Notch system is dynamically regulated, and that blocking one Notch ligand increases the effective concentration of other Notch ligands, thus altering the response. Examination of genes related to the Notch pathway revealed that the Notch receptors were increased in memory T cells. Expression of BMI1, a gene involved in T cell proliferation, was also higher in memory T cells. Further experiments demonstrated that Notch directly regulates the expression of the BMI1 gene in T cells and may govern T cell proliferation through this pathway.

**Conclusions:**

From these experiments we can make several novel conclusions about the role of Notch ligands in T cell biology. The first is that delta-like 4 suppresses effector cell proliferation and enhances Th2 memory cell proliferation. The second is that blocking one Notch ligand in vivo effectively increases the concentration of other Notch ligands, which can then alter the response.

## Introduction

The Notch system consists of 4 receptors and 5 ligands that interact to direct cell fate [1]. These interactions are important during the growth and development of an organism. It is known that Notch is also critical in directing T cell responses in both Th1 and Th2 settings[2], [3], [4], [5]. Furthermore, it has been hypothesized that differential Notch ligand expression by dendritic cells (DC) can help skew a T cell response towards a Th1 or Th2 phenotype[6]. While it is known that the inducible notch ligand delta-like 4 can decrease Th2 cytokines during a primary viral response [7], the role of notch ligands in established Th2 responses has not been studied.

It is generally accepted that memory T cells, upon encountering antigen, are quick to proliferate and are poor cytokine producers. Effector cells, on the other hand, are poor proliferators but produce effector cytokines efficiently [8], [9], [10]. The gene expression patterns of effector cells are also very different than that of memory cells, and their behavior *in vivo* corroborates these differences[11]. Several studies have shown that different Notch ligands can have opposing effects on T cell biology, but no studies have examined the different response of memory and effector T cells to Notch ligands [2], [12]. We used a secondary Th2 response to examine the role of Notch ligands in Th2 cell biology. Our data indicate that effector and memory Th2 cells respond differently to the ligand delta-like 4. We found that while effector cell proliferation is suppressed by delta-like 4, Th2 memory cell proliferation is enhanced by this same molecule. We demonstrate that the protein BMI1, which is involved in many aspects of T cell biology including proliferation and survival, is also expressed at higher levels in memory cells relative to the level found in effector cells[13], [14]. Furthermore, our experiments reveal that memory T cells and their progeny have increased expression of the Notch receptors on their cell surface. Our data indicate that the delta-like 4 signal controls T cell proliferation by directly influencing the transcription of BMI1. Thus we reveal a novel mechanism through which Notch regulates the proliferation and survival of the effector and memory cell subsets.

## Materials and Methods

### Mice

Experiments were done in the C57 Bl/6 strain. All mice were purchased from Taconic (Germantown, NY) and were between 6 and 8 weeks old at time of sensitization. All experiments were done with the approval of the University of Michigan Committee for Use and Care of Animals (UCUCA) under protocol 8307 (approval dates 11/26/07–11/26/10).

### Generation of polyclonal antibody

Rabbit anti-murine jagged-1 and anti-delta-like 4 antibody were prepared by multiple-site immunization of New Zealand White rabbits with recombinant protein (R&D Systems, Rochester, MN) in CFA and boosted with recombinant protein in IFA as in previously described procedures from our laboratory [7]. Polyclonal antibodies were titered by direct ELISA against the appropriate protein coated onto 96-well plates. Serum from unimmunized rabbits was used for a control treatment group. Antibody specificity was verified by western blot against OP-9 cell lines expressing Notch ligands (provided as a generous gift from Dr. John Lowe)[15].

### Western Blotting

Western blotting was performed using standard techniques. To blot for jagged-1 we used a non-reducing sample buffer and a 10% acrylamide gel. Protein A purified anti jagged-1 antibody was used at a dilution of 1∶1000. Specificity was determined by using lysates from OP-9 cells stably transfected with Notch ligands. For BMI1 detection a rabbit polyclonal BMI1 antibody from Abcam (Cambridge, MA) was used. A Femto detection kit from Pierce (Rockford, IL) was used for film development of BMI1 blot.

### S. Mansoni eggs

S. Mansoni eggs were purified as previously decribed [16]. Live S. Mansoni eggs were purified from the livers from mice heavily infected with S. Mansoni, (mice provided by Dr. Fred Lewis (Biomedical Research Laboratory, Rockville, MD)).

### Measurement of cytokines

IL-4 was measured using Bioplex kits from Biorad (Hercules, CA).

### Cell sorting and transfer

Cells were sorted on a BD FACS Vantage. Prior to transfer, cells were obtained from the spleens of 5 mice 6 weeks post immunization. Single cell suspensions were made of the splenocytes, red blood cells lysed, and subjected to negative selection using a MACS column with a cocktail of antibodies against CD8, CD19, and MHC-II (Miltenyi, Auburn, CA). Cells were then stained with anti CD4, anti CD44, anti CD62L and anti CCR7 after incubation with anti CD16/32 (FC block). To sort cells we used the following antibodies (all purchased from eBioscience): CD44 (clone IM-7), CD4 (clone RM4-4), CD62L (clone MEL-14), and CCR7 (clone 4B12). All antibodies were purchased from eBioscience (San Diego, CA). After cells were sorted they were injected intravenously into S. Mansoni sensitized mice that had been injected intravenously with S. Mansoni eggs 2 hours prior to receiving donor cells. A typical experiment would result in the transfer of 200 memory cells/mouse or 70,000 effector cells/mouse.

### Flow cytometric analysis

Lungs were minced and digested with 1 mg/mL Collagenase A (Roche Applied Science) in RPMI containing 10% FCS for 45 minutes. The suspension was then passed through 5 mL syringe with an 18G needle 20x, RBCs lysed, and then passed through a 100 µM nylon mesh. Single cell suspensions were also made from lymph node cells. All staining was done in a 96 well plate with 3x washes after antibody incubation. Cells were fixed in 20 µL 5% formalin and resuspended in 200 µL PBS before analysis. Cells were analyzed on a LSR II equipped with a 488 nm, 633 nm, and 405 nm laser (BD, San Jose CA). Antibodies to Notch receptors 1–4 were purchased from Biolegend (San Diego, CA).

### Proliferation assay

Single cell suspensions were made from the splenocytes of immunized mice, and RBCs lysed. 1.0×10^6^ cells were loaded in 100 µL/well of a 96 well plate. Recombinant Notch ligands were purchased from R and D (Minneapolis, MN) and coated on the plate in 100 µL PBS at 37°C for 2 hours prior to loading cells. Soluble egg antigen (SEA) was given at a concentration of 10 µg/mL. Cells were allowed to proliferate for 4 days. On the fourth day, cells were pulsed with 1 µCi of ^3^H thymidine for 5 hours. Cell DNA was then collected on a PhD harvester (Gaithersburg, MD) and counted in a scintialltion counter. There was no difference above background when cells were exposed to Notch ligands in the absence of antigen.

### Naïve CD4^+^ T cell isolation and stimulation

Cells were taken from whole spleens, FC blocked, and incubated with the following antibodies: DX5, CD8, CD19, gamma delta, Ter119, CD11b (eBioscience). After washing, anti-biotin beads (Miltenyi) were added to the cells and the cells were then run through an LS negative selection column (Miltenyi). Cells were then stimulated for 4 hours with 5 µg/mL of plate-bound antiCD3 and 3 µg/mL of soluble antiCD28 (BD Biosciences, San Jose, CA), and 10 nm of plate-bound jagged-1 or delta-like 4 (R&D, Minneapolis, MN). The plates were coated for 2 hours before cells were added.

### RNA isolation and analysis

Data from RNA analysis relating to BMI1 expression in memory and effector cells was obtained by isolating RNA using the Qiagen micro isolation kit (Valencia, CA). RNA was then amplified using an RNA amplification kit from Ambion (Austin, TX). 20 ng of amplified RNA was then subjected to gene specific reverse transcription using Superscript III from Invitrogen (Carlsbad, CA) and primer sets from Applied Biosystems (Carlsbad, CA). We then used the same primer probe sets to analyze the level of gene transcription by Taqman. Analysis was performed by taking the inverse of the difference between the control gene cycle number and the gene of interest cycle number. In the experiment relating to BMI1 expression in memory and effector cells, the control gene used was murine Rplp2 (probes obtained from Applied Biosystems). This gene was shown to have a minimal amount of variation among human leukocytes [17]. For analysis of naïve T cell mRNA we used 1.0×10
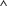
5 cells/well of a 96 well plate and isolated RNA by Trizol (Invitrogen). For all experiments assessing BMI1 RNA, the primers for Taqman analysis by SYBR green were as follows:

F: CCAGACCACTCCTGAACATAAGG

R: AAGCCCTGGGACTAATTTGTATACA

### ChIP Assay

Methods followed previously described protocol [18]. We used an anti mouse RBPJK monoclonal rat antibody from Cosmo Bio Co., Ltd (Tokyo, Japan). Custom designed primers flanking the RBPJK binding site of the BMI1 promoter had the following sequence:

F primer - GAAACCCTACTGGGAAGGTAGGTAC

R primer - GGGTCAGGATCCCAATTTTT

Primers were purchased from Sigma- Aldrich (St. Louis, MO)

## Results

### Memory and Effector T cells subsets respond different to Notch ligands *in vitro*

**Figure 1:**
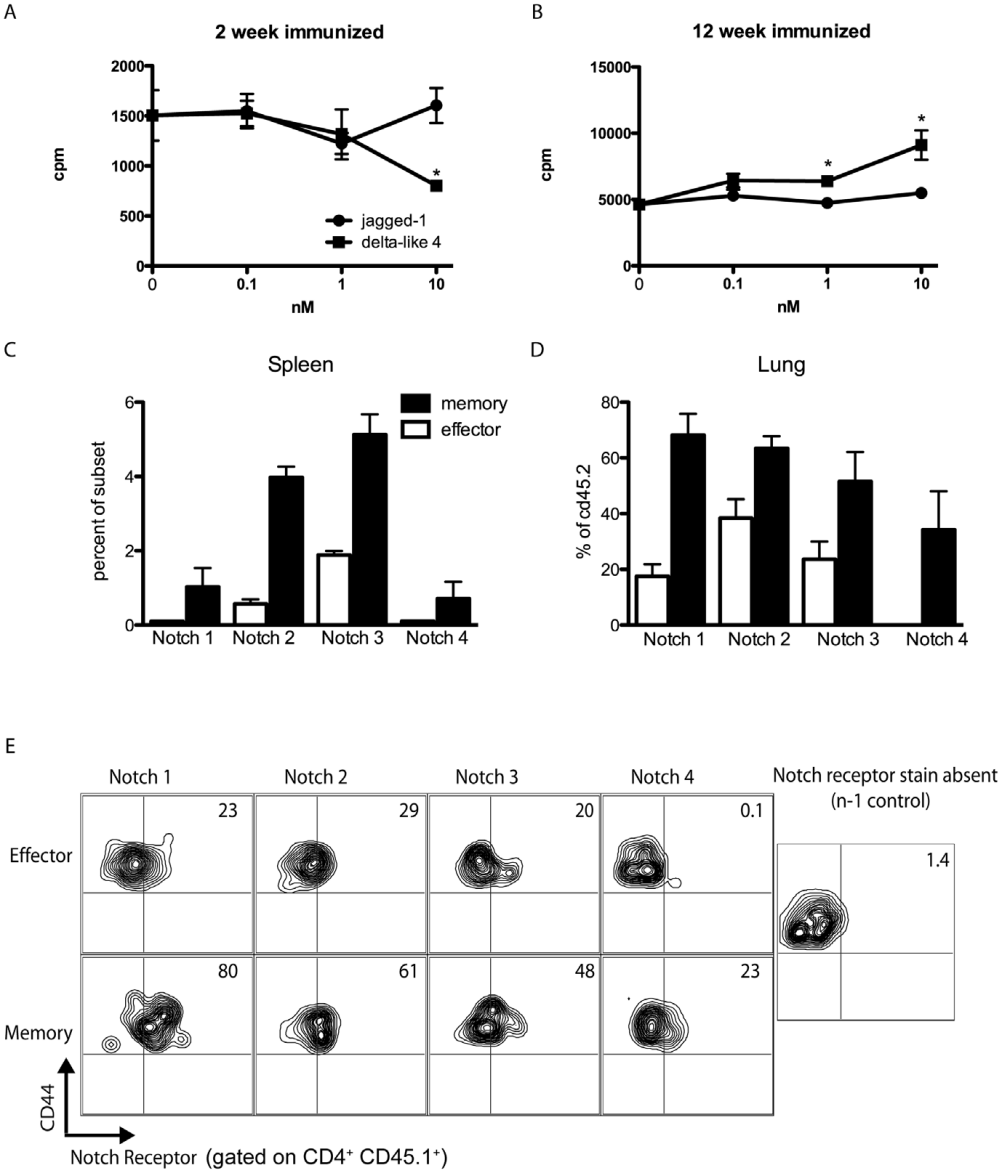
Memory and effector cells have different Notch related responses and express different levels of Notch receptors. A,B) Splenocytes from a mouse either 2 weeks or 12 weeks post S. Mansoni egg immunization were restimulated *in vitro* with SEA and increasing concentrations of delta-like 4 or jagged-1. *p<0.05 C) Percent of endogenous memory or effector cells in the spleens of mice immunized for 6 weeks with S. Mansoni eggs. All cells expressing memory cell markers expressed a higher level of Notch receptor than those cells expressing effector cell markers (p<0.05) D) Percent of donor (CD45.2) population expressing Notch receptors after incubation in a congenic sensitized mouse receiving S. Mansoni egg challenge. In all cases, the percentage of donor derived cells from those mice receiving memory cell transfer expressing a given Notch receptor were significantly higher than donor derived cells from mice receiving effector cell transfer. (p<0.05) E) Flow plots gated on either effector or memory donor (CD45.1^+^) cells at day 9 of the secondary S. Mansoni egg model.

As Notch is involved in both the development and skewing of T cells, we hypothesized that Notch may also have a role in the effector and memory CD4 T cell response. To determine the possible role of Notch on these subsets we stimulated whole splenocytes from mice sensitized with S. Mansoni eggs for two or 12 weeks with soluble egg antigen (SEA) and varying concentrations of Notch ligands and measured both proliferation and cytokine output. Splenocytes from a mouse immunized for two weeks will generate largely an effector cell response, whereas splenocytes from mice immunized for 12 weeks will contain a memory cell population specific for the SEA. We found that while jagged-1 had no effect on the proliferative capacity of either group, delta-like 4 had opposing effects on effector and memory CD4^+^ T cell responses. High concentrations of delta-like 4 inhibited effector cell proliferation, but increased the proliferative capacity of memory cells (Fig. 1 a and b).

We next performed flow cytometry to examine if the difference in response between these two subsets was due to the expression of a different repertoire of Notch receptors. We first examined the spleen of 6 week immunized mice and found that there were higher levels of all Notch receptors expressed on memory cells (CD4^+^ CD44^hi^ CD62l^+^ CCR7^+^) than effector cells (CD4^+^ CD44^hi^ CD62l^−^) (Fig. 1c). It was necessary to perform a congenic transfer experiment to ascertain if the expanded memory cell population, or secondary effectors, also expressed increased levels of Notch receptors when compared to primary effectors as these cells look identical to primary effector cells after expansion in terms of CD44 and CD62L expression. For these experiments we isolated CD45.1 memory cells or effector cells from 6 week S. mansoni immunized mice and transferred into CD45.2 S. mansoni immunized mice and again used flow cytometry to determine the level of Notch receptor expression on the expanded donor cell population (1d–e). Regardless of whether the donor cells were of memory or effector phenotype, the majority of cells that were CD45.1^+^ were of the effector phenotype (CD44^hi^CD62L^lo^) at the time of analysis. Thus the transferred effector cells maintained a similar cell marker phenotype as when they were transferred in, and memory cells isolated as CD44^hi^CD62l^+^CCR7^+^ differentiated into secondary effector cells (Figure S1). Surprisingly, we found that the expanded memory cell population also expressed a higher level of Notch receptors than primary effector cells.

### Blocking Notch ligands in vivo alters the Th2 response

**Figure 2:**
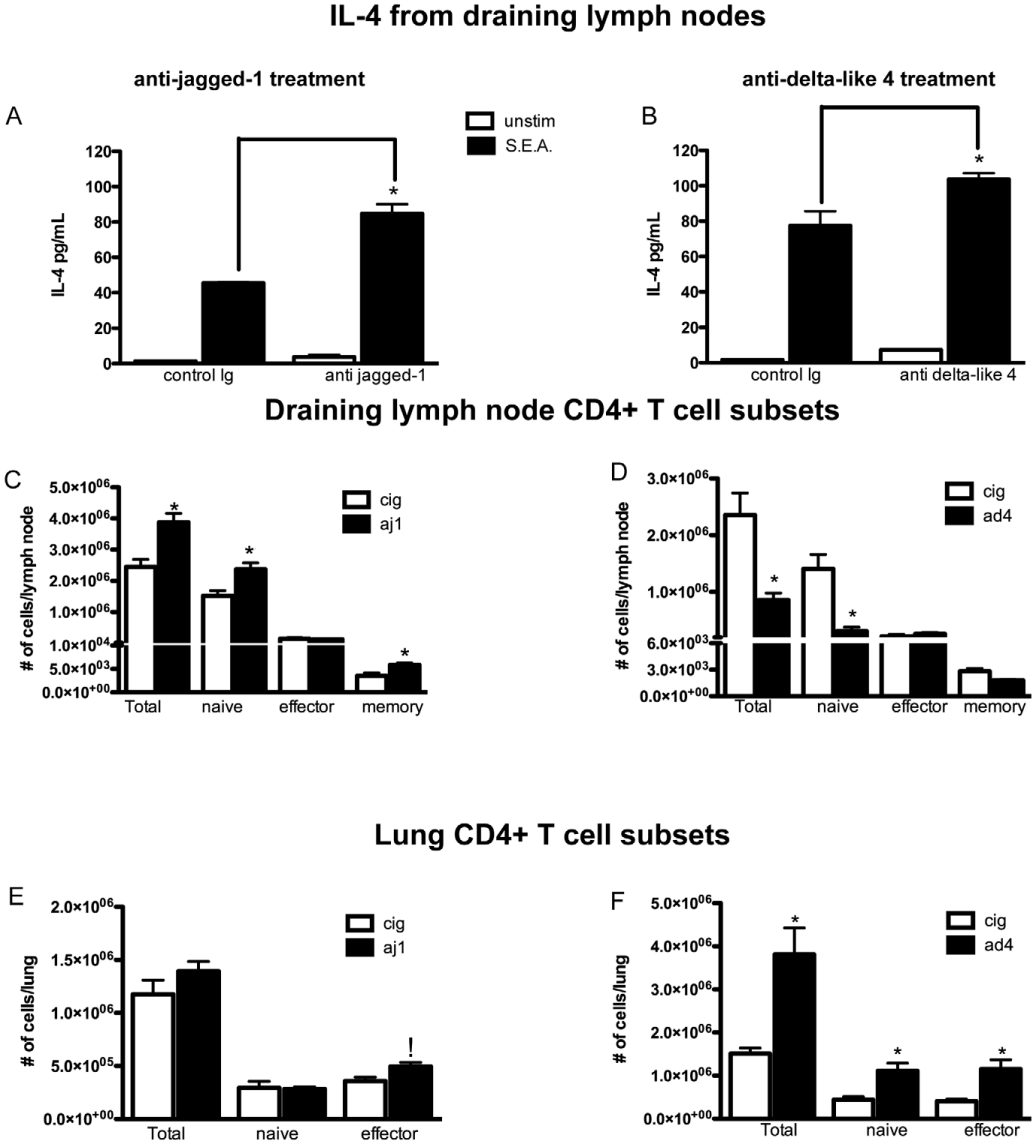
Anti jagged-1 and anti-delta-like 4 treatment alter the Th2 immune response. A,B)Lymph node cells from mice treated with anti-notch ligand antibodies produced significantly more IL-4 when restimulated with SEA. *p≤0.01 using one-way ANOVA C–F) Changes in CD4^+^ T cell populations in the lung and lymph node during secondary S. mansoni immune response with anti-Notch ligand treatment. *p≤0.05 using unpaired T test.

To determine if jagged-1 and delta-like 4 were important in Th2 responses we used a model of chronic Th2 inflammation induced by a 14-day sensitization to *S. mansoni* eggs followed by an intravenous challenge with these same eggs. In separate experiments we administered a specific polyclonal antibody to jagged-1 or delta-like 4, as previously characterized [7], every other day during the challenge phase of the model. Our antibodies were shown to be specific and function to block the ligand *in vivo* (Fig. S2). On day 8, lungs and lymph nodes were taken from mice for analysis (Figure 2). Restimulation of cells from the draining cervical and mediastinal lymph nodes with schistosoma egg antigen (SEA) showed that those mice receiving anti Notch ligand produced higher levels of IL-4 (Fig. 2a–b) with both treatments. Flow cytometric analysis revealed a number of changes in the population of lung and lymph node T cell subsets (Fig. 2c–f)[19], [20]. Interestingly, the number of effector cells in the lymph nodes of mice treated with anti Notch ligand antibodies remained the same as in control mice. However, in both anti-jagged-1 and anti-delta-like 4 treated mice there was an increase in effector cells in the lung. In mice receiving either anti delta-like 4 or anti jagged-1 treatment, there was no difference observed in the number of B cells (CD19^+^) or CD8^+^ T cells (Fig. S3). There was also no difference in the number of non-viable cells in any CD4^+^ T cell subset, indicating this increase in effector cells was not due to increased cell death.

### Jagged-1 and delta-like 4 control memory and effector T cell proliferation

As we had previously characterized the role of delta-like 4 in a T cell driven primary response [7], we also sought to test the possible role of jagged-1 in an unsensitized mouse. To test if the changed observed in CD4^+^ cells was specific to previous sensitization with antigen or could be observed during the primary immune response, we injected S. mansoni eggs into mice that had not been previously sensitized. We found that with this model there was no increase in IL-4 production from restimulated lymph nodes when comparing anti jagged-1 treated mice to controls (Fig. S4). Moreover there was a decrease in the number of total and effector cells found in the lung and an increase in the number of effector cells found in the lymph node (Fig. S4b–c). These data indicate that jagged-1 blockade does not have the same effect in a primary model of inflammation as it does during an anamnestic response. Because of the change in effector cell number found in this primary response model, we wanted to investigate if this was an antigen specific phenomenon. We thus transferred naïve OTII CD45.2^+^ cells into a CD45.1 mouse and challenged with OVA. All CD4^+^CD45.2^+^ cell were CD44^hi^ (Fig. S4d), thus indicating that they had been activated. However, we found that there was no increase in antigen specific effector cells during a jagged-1 blockade, thus demonstrating that jagged-1 has no role in the conversion of naïve to effector cells (Fig. S4 e–f). From these data we concluded that jagged-1 blockade does not have the same effect in a model of primary inflammation as it does during an anamnestic response. Furthermore, anti-jagged-1 treatment does not decrease the conversion of antigen specific naïve CD4^+^ T cells to effector cells.

**Figure 3:**
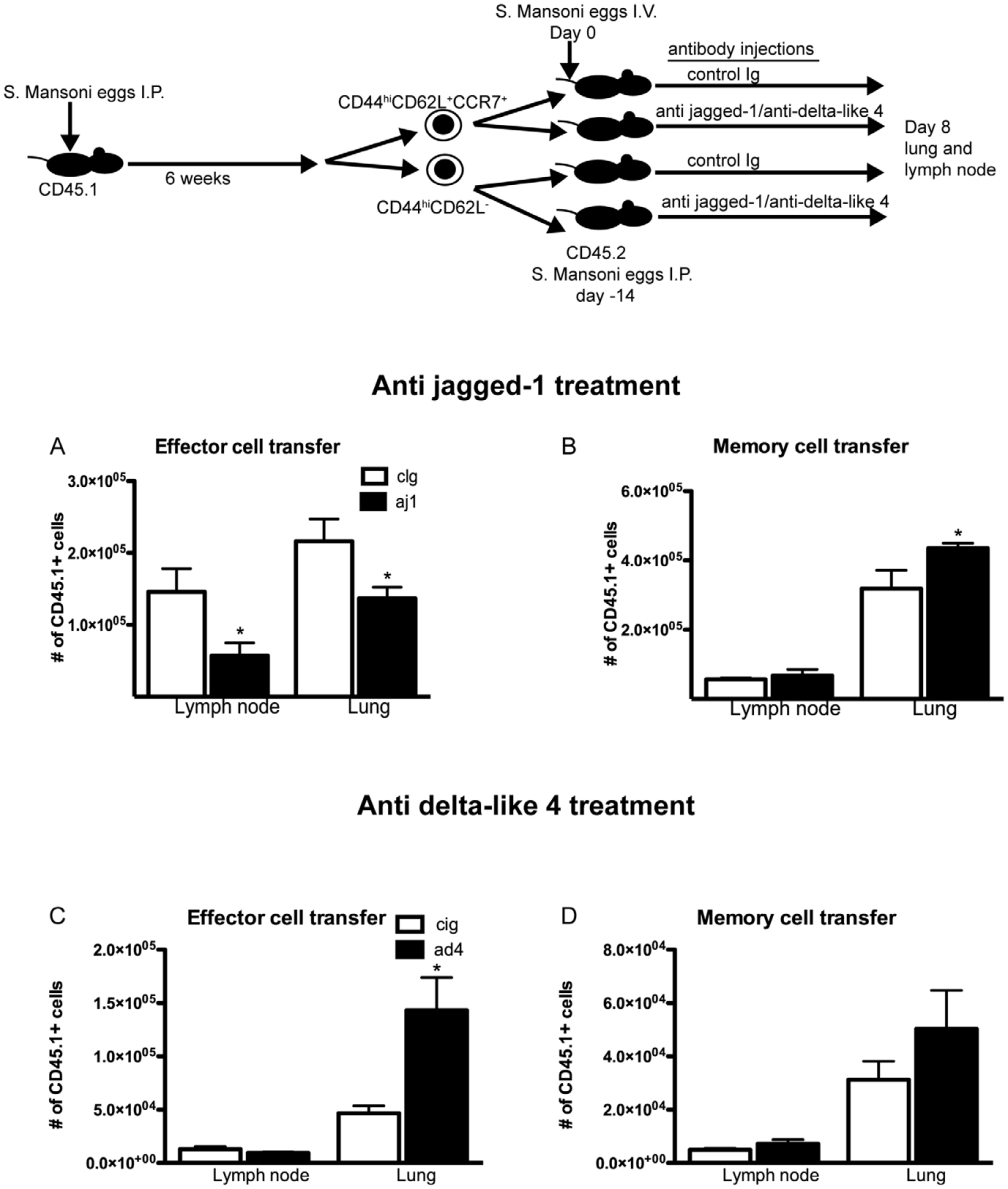
Memory and effector cell expansion are differentially affected by Notch ligand blockade *in vivo*. Congenic effector or memory cells were transferred from S. mansoni sensitized donors into similarly sensitized hosts that subsequently received S. mansoni egg challenge A,B) Effector cell number was reduced in the presence of anti jagged-1 in both the lung and the lymph node, whereas the same treatment increased the proliferation of secondary effectors (derived from memory cells) in the lung. C,D) Anti delta-like 4 treatment during the same response causes a significant increase in the amount of effector cells. *p≤0.05 in all cases using an unpaired T test analysis.

Because there was no increase in the expansion of naïve T cells in mice given a jagged-1 antibody, we hypothesized that the observed increase in effector cells in previously immunized S. Mansoni challenged mice treated with anti Notch ligand antibodies arose from alterations in the proliferation of T cells previously exposed to antigen. To determine the source of the increase in effector cells that occurred in our anamnestic immune model, we immunized CD45.1 mice with S. mansoni eggs for 6 weeks and isolated CD4^+^ memory and effector cells based on expression of CD44, CD62L and CCR7. Effector and memory cells were then transferred into separate S. mansoni sensitized hosts that were given an intravenous challenge of S. mansoni eggs to induce lung inflammation. While a sizable population of congenic T cells was found in the lung and lymph node, there were very few found in the spleen. The majority of congenic T cells in both the lung and lymph node expressed markers consistent with an effector cell phenotype (demonstrated in Figure S1). Consistent with previous reports, memory cells had a large capacity for expansion whereas effector cells were poor proliferators[21]. We found that the proliferative capacity of these subsets was significantly altered by treatment with antibodies to notch ligands (Fig. 3). While memory cells increased their proliferative capacity in the absence of jagged-1, the expansion of effector cells was reduced under the same conditions. Conversely, anti delta-like 4 increased effector cell yield and had no effect on the expansion of memory cells.

These data corroborate our *in vitro* findings shown in Figure 1, which demonstrate that supplying delta-like 4 to effector and memory cell populations can have opposite effects on cell proliferation. We argue that by blocking one of the two ligands *in vivo*, access to the other ligand is effectively increased, thus altering the T cell response by increasing or decreasing the rate of expansion of the memory or effector cell population. We performed additional studies using an OVA/alum model and antigen specific OTII cells, and obtained the same results with anti jagged-1 as shown in Figure 3, thus suggesting this is an antigen specific phenomenon (Fig. S5a). We also performed a set of transfer experiments in a Th1 driven response. These studies show a similar decrease in effector cell yield when jagged-1 is absent, but no change in the amount of secondary effector cells derived from memory cells after jagged-1 blockade (Fig. S5b). From these studies we can conclude that the decrease in effector cells observed with jagged-1 blockade to be an antigen specific phenomenon that occurred in both Th1 and Th2 inflammation. The increase in memory cell expansion that occurred with anti jagged-1 blockade was specific only to Th2 memory cells.

### BMI1 is regulated by Notch

**Figure 4:**
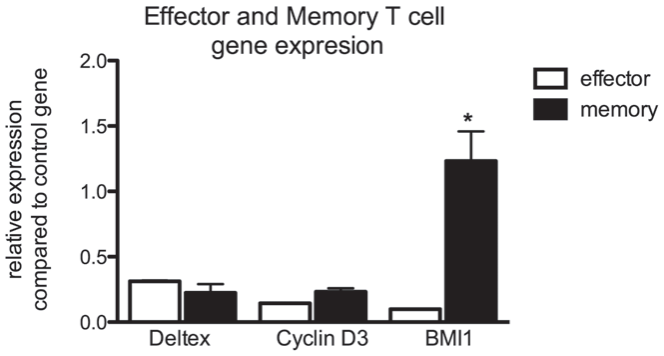
BMI1 expression is higher in memory cells than effector cells. Memory and effector cells were isolated from animals immunized for 6 weeks with S. Mansoni eggs. RNA was isolated, amplified, and quantitative PCR performed to assess the expression of genes associated with Notch signaling or proliferation. Results are expressed relative to a control gene (Rplp2) that has been shown to have minimal variation in leukocytes. *p≤0.01.

To investigate how increased Notch signaling might increase the proliferative capacity of memory cells, we examined the expression of several genes that are regulated by the notch pathway or associated with the proliferation of T cells. For these experiments, memory or effector T cells were isolated from the spleens of mice sensitized to S. Mansoni eggs 6 weeks previously. RNA was then extracted and amplified from these cells, and assayed by real time PCR. Many genes regulated by the Notch pathway were expressed in effector cells, but not in memory T cells. These included the fringe family of genes as well as hes5. Expression of the cyclinD3 gene, which is described as being regulated by the Notch pathway, was expressed in both memory and effector T cells at similar levels [22]. The same was true of the Notch regulated gene deltex 1. We found that the gene that was most highly expressed in memory T cells relative to the amount expressed in effector cells was BMI1 (Fig. 4). It is known that this gene is important for T cell proliferation and survival of memory T cells [13], [14]. Furthermore, this protein stabilizes the GATA3 protein thus promoting a Th2 phenotype [23].

**Figure 5:**
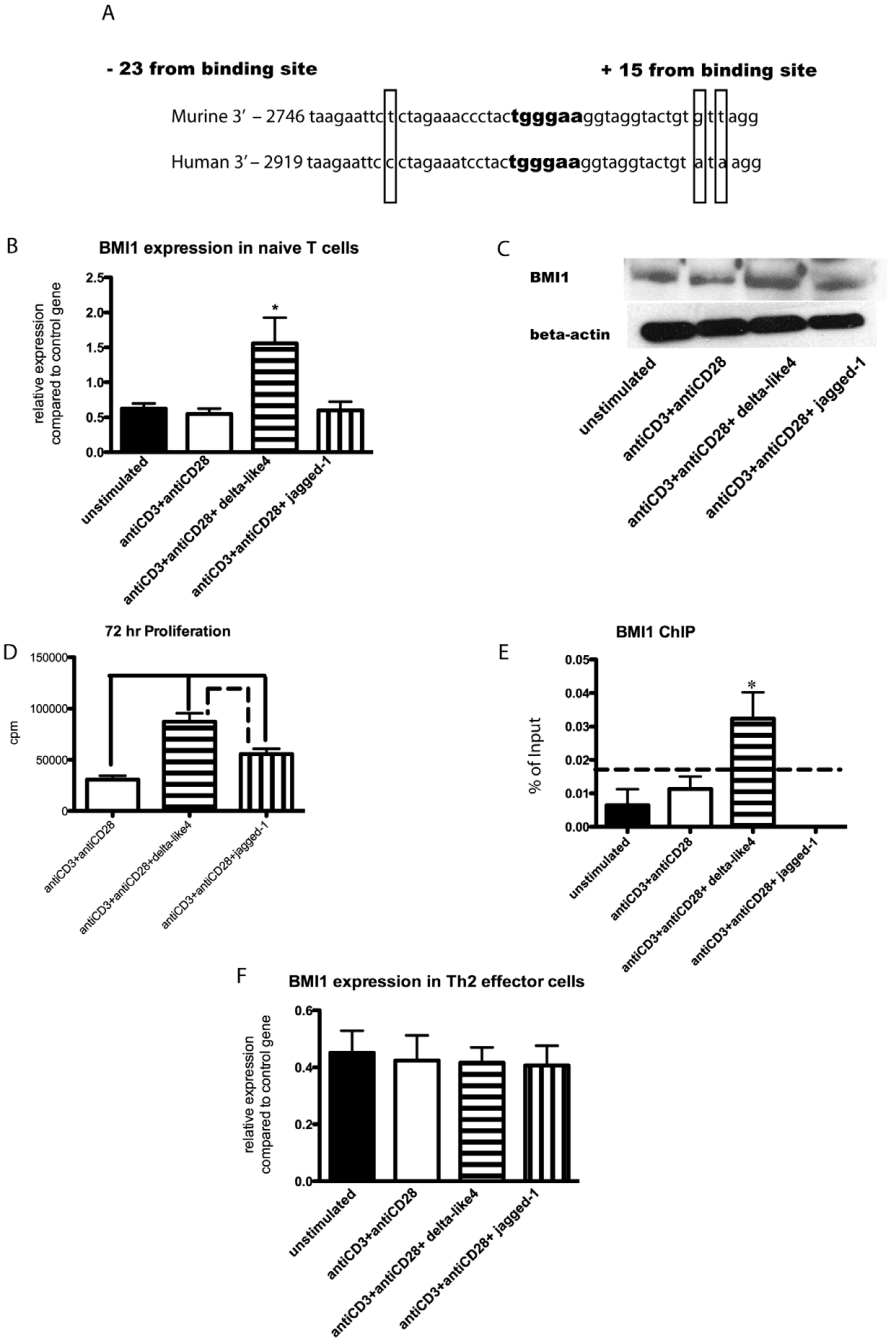
Delta-like 4 increases BMI1 expression in naïve T cells. A) Alignment of nearly homologous regions of mouse and human BMI1 promoter sequence with RBPJK binding site highlighted. B) BMI1 RNA expression in naïve T cells with treatment groups antiCD3/antiCD28, antiCD3/antiCD28+ delta-like 4, and antiCD3/antiCD28+ jagged-1. *p<0.05. C) Western blot protein data with same treatment conditions as B. D) Proliferation assay with same treatment conditions as B. Delta-like 4 v. CD3, p = 0.0001; delta-like 4 v. jagged-1, p = 0.0096; jagged-1 v. CD3, p = 0.0031. E) ChIP assay measuring binding of RBPJK to BMI1 promoter under same treatments as B. The dotted line represents the level of background obtained from using a non-specific control antibody. *p<0.05. F) RNA levels of BMI1 fromTh2 effector cells after stimulation with Notch ligands and CD3/CD28 stimulation.

We found through a separate screen that BMI1 was regulated by Notch ligands in naïve T cells. Thus we used naïve T cells to investigate the role played by the Notch signaling pathway in BMI1 expression. Initially, we located an RBPJk binding site in the BMI1 promoter region of mice and a nearly homologous region in humans approximately 2.7 kB from the transcriptional start site (Figure 5a). Next, we investigated whether or not Notch ligands do in fact affect BMI1 expression by stimulating naïve T cells with anti-CD3/anti-CD28 in the presence of plate bound delta-like 4 or jagged-1. Analysis of mRNA revealed that the level of BMI1 was increased in cells that had been exposed to delta-like 4 compared to unstimulated cells and cells stimulated with anti-CD3/anti-CD28 alone or in the presence of jagged-1 at four hours post stimulation (Figure 5b). This RNA data was confirmed by Western blot, which also showed an increase in the protein level of BMI1 in cells treated with delta-like 4 at 24 hours post stimulation (Figure 5c). Next, we looked at the effect delta-like 4 has on naïve T cell proliferation. We found that cells treated with delta-like 4 demonstrated an increased proliferation, while jagged-1 had no effect on the proliferative capacity of the cells (Figure 5d). Together, these results suggest that BMI1 expression is upregulated by delta-like 4, and that BMI1 works to augment the proliferative capacity in naïve T cells. We also performed a ChIP assay to determine if RBPJk binds to the promoter region of BMI1. To do this, we used an RBPJk antibody, and designed primers flanking the binding site for RBPJK in the BMI1 promoter region. In accordance with all our other results, we found that there is significantly more RBPJk binding to the BMI1 promoter region in naïve T cells stimulated with anti-CD3/anti-CD28 and delta-like 4 compared to any of the other groups (Figure 5e). We also wanted to determine if Notch regulated effector cell expression of BMI1. To do this we isolated effector cells from the spleens of S. Mansoni sensitized mice in the same manner as in Figure 3 and stimulated those cells with recombinant Notch ligands in the presence of anti-CD3 and anti-CD28. We found that in the effector cell population, Notch ligands did not upregulate BMI1 expression (Figure 5f). Furthermore, we did not find regulation of BMI1 by delta-like 4 in Th1 effector cells. As BMI1 expression has been linked to suppression of the Noxa gene [13], we analyzed naive CD4 T cells stimulated with delta-like 4 to determine if Noxa expression was altered. Figure S6 demonstrates that naïve cells have a reduction in Noxa expression when exposed to delta-like 4. This data correlates with previously published results on the mechanism for BMI1 mediated survival of T cells, and suggests that BMI1 is functional in naïve T cells.

## Discussion

### Delta like-4 and Jagged-1 alter the proliferative capacity of T cells

The adaptive immune response is a tightly controlled system that can be harmful to the host when dysregulated. It is known that the Notch system is important in determining the fate of a T cell in terms of both proliferative capacity and the bias of a cell towards a Th1 or Th2 phenotype [3], [4]. The role of this system in the regulation of the effector and memory response has not previously been investigated. Our findings are contrary to the current models of how Notch is integrated into T cell biology [6]. We demonstrate that effector and memory cell populations respond differently to jagged-1 and delta-like 4. While effector T cells, regardless of their Th1 or Th2 bias, require jagged-1 for normal proliferation regardless of the immune response, Th2 memory cells specifically expand more in the presence of delta-like 4 than with jagged-1. We demonstrate by blocking one ligand or the other in congenic transfer models, that the normal immune response is dependant on both ligands being present at the same time to dictate the balance of memory and effector cell expansion. In this system, two separate cell populations require different Notch signals to achieve the same effect. While exposure of effector T cells to delta-like 4 causes repression of proliferation, exposure of the memory cells to the same ligand causes an increase in their proliferative capacity. The increase in effector cell number in anti jagged-1 treated mice is due to an increase in central memory cell proliferation and differentiation into effector cell phenotype. However, the increase in effector cell number in anti delta-like 4 treated mice is due to an increase in effector cell expansion. The increase in effector cell proliferation that occurs with anti delta-like 4 treatment is likely correlated to the suppressive effects that we observed upon exposure of effector cells to recombinant delta-like 4 *in vitro*.

### Primary vs secondary effector cells

It is well known that memory lymphocytes expand upon exposure to an antigen previously encountered by the immune system. We demonstrate here that the progeny of memory cells, although expressing similar markers to those of primary effectors in terms of CD44, CD62L and CCR7, have altered expression of Notch receptors similar to that seen in splenic memory cells. Interestingly, we did not find a dominant receptor expressed on memory cells. Instead, these cells seemed to express a higher level of every Notch receptor, including an expression of Notch 4, which is completely absent on effector cells. This finding has a number of implications. The first is that it may be possible to distinguish a primary from a secondary effector cell in a given response. More importantly, targeting the Notch pathway may give us a way to enhance or inhibit an effector T cell response without inhibiting the memory T cell response, as memory T cells are more resistant to disruption of Notch signaling. The changes that take place during the contraction phase of a given immune response require further study in order to determine when memory cells acquire the ability to express higher levels of Notch receptors. As the increase of Notch receptor expression appears to last through the expansion phase of memory cells to secondary effectors, high levels of Notch receptors may end up being a way to determine if a host response is secondary or primary in nature.

### Notch signaling augments T cell proliferation

Our data indicate that isolated memory cells express high levels of the RING finger protein BMI1 when compared to effector cells. However, both effector and memory populations express the cell cycle gene cyclin D3. Both genes have previously been shown to be essential to T cell proliferation [13], [22]. Here we show that BMI1 is upregulated by the exposure of T cells to delta-like 4. Our data definitively show that delta-like 4 causes enhancement of the proliferative capacity of T cells and binding of RBPJK to the BMI1 promoter. Studies have also indicated a role for BMI1 in promotion of Th2 cell differentiation via the stabilization of GATA3 [23]. While BMI1 expression and Notch activation have been correlated in the thymus [24], it is a novel finding that the Notch pathway directly regulates BMI1. Mice deficient in delta-like 4 in the thymus have a reduced number of double-positive T cells ([25] and Fig. S2). Mice lacking BMI1 also have a deficiency in the number of pre-T cells that transition form double negative to double positive [26]. It is likely that the defect described upon deletion of delta-like 4 is due in part to a failure of pre-T cells to up regulate BMI1 during the transition from double negative to double positive thymocytes.

The binding of RBPJ-K to the BMI1 promoter only occurs in the presence of delta-like 4, indicating that delta-like 4 and jagged-1 do not send the same signal. Interestingly, primary effector cells show no regulation of BMI1 expression at the RNA level in the presence of delta-like 4. Thus our data indicate that the delta-like 4 ligand can have a different effect on the effector subset compared to either naïve or memory cell populations. While supplying delta-like 4 *in vitro* can increase memory cell proliferation, it decreases the proliferation of the effector cells specific for the same antigen. We also demonstrate *in vivo* that delta-like 4 suppresses the proliferation of effector cells by performing congenic transfers and blocking this ligand, which increases effector cell yield from the congenic host (Fig. 3). It is possible that the regulation of the BMI1 promoter in memory cells by the ligand delta-like 4 provides a way for the immune system to quickly reactivate the memory response to a foreign antigen, while at the same time suppressing the ongoing effector response.

### Notch ligands contribute to a balanced immune system

Our data indicate a number of novel findings regarding the relationship of the Notch pathway to T cell biology. These findings illustrate the complexity of T cell biology and the degree of fine-tuning done by the immune system to create a specific response to a foreign antigen. Our results demonstrate that while treatment with both anti jagged-1 and anti delta-like 4 increases effector cell number, the cause of this increase is different in each treatment group.

**Figure 6:**
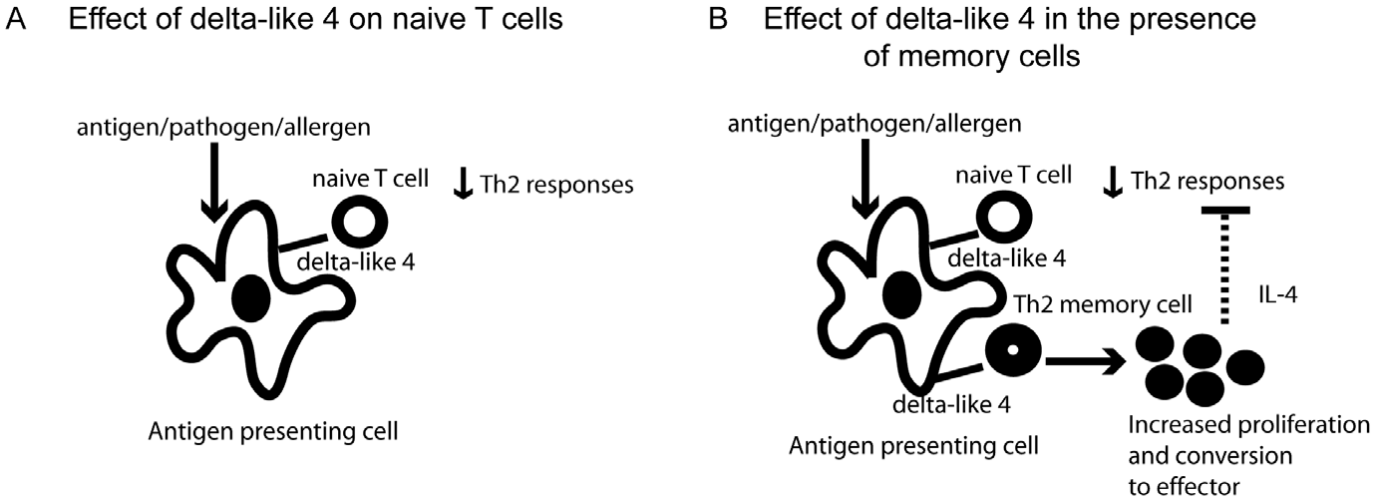
Possible mechanism by which delta-like 4 regulates the T cell immune response.

*In vivo* the immune system may use the inducible ligand delta-like 4 to preferentially expand the Th2 memory T cell subset when it encounters an antigen. This is consistent with genetic manipulations of the notch pathway demonstrating that Th2 responses are dependant on Notch [2], [4]. This is in contrast to what happens in a primary response, where delta-like 4 is used to suppress Th2 cytokine production [7]. By rapidly proliferating in response to delta-like 4, Th2 memory cells may be able to direct the response in an antigen specific manner even as naïve cells are responding to the same antigen and notch ligand by suppressing Th2 cytokines. It is known that memory cells can proliferate more rapidly and have a lower threshold of activation than naïve cells [27]. Thus the expression of delta-like 4 may give the immune system an option to respond to an antigen with Th1 or Th2 cytokines (summarized in Figure 6). This option would depend on upregulation of delta-like 4 on the dendritic cells as well as if the immune system has mounted a response to that antigen at some point previous to the current encounter.

While it is known that delta-like 4 can be induced in a MyD88 dependant manner, it is not known what induces delta-like 4 expression in a Th2 model. Recently, schistosoma egg antigen has been shown to induce the expression of jagged-1 on dendritic cells *in vitro* via a TLR2/4 dependant mechanism[28]. Since delta-like 4 is MyD88 dependant, and has been shown to be upregulated upon exposure of dendritic cells to LPS[29], egg antigen may cause delta-like 4 to be expressed on dendritic cells as well. Other Th2 antigens, such as OVA and cockroach antigen, have also been shown to initially signal through the MyD88 pathway [30], [31]. Thus a possible mechanism by which Th2 memory cells proliferate is through the MyD88 dependant expression of delta-like 4, which is a pathway that has already been demonstrated as important in a number of studies [3], [29]. If the effective concentration of delta-like 4 is increased by jagged-1 blockade, then a greater secondary Th2 response should be observed due to increased memory cell activation and proliferation. We found this to be true as more IL-4 was produced upon blockade of jagged-1 (Fig. 2a).

The subsets of CD4^+^ T cells are many and varied, and it is likely that delta-like 4 regulates all of them in some way. Delta-like 4 has previously been shown to be important in the generation of both Th1 and Th17 cells [3], [32], [33]. Here we show that it is also important in the activation of Th2 memory cells. Because three separate subsets of the same cell use delta-like 4 as an activating signal, this molecule is clearly important in generating an immune response. We also show a role for the maintenance of effector cell populations by the ligand jagged-1. By demonstrating opposing roles for the notch ligand delta-like 4 on effector and memory populations we have defined a new role for Notch in the immune system. Our data suggests that jagged-1 is used as a ligand to maintain the current immune response by maintaining effector cell proliferation. Hence, if the effective concentration of jagged-1 is increased with delta-like 4 blockade, then suppression of effector cell proliferation should be reduced and a greater Th2 response should be observed. We found more IL-4 production with anti delta-like 4 blockade upon antigen restimulation (Fig. 2b) and a greater number of primary effector cells were recovered upon anti delta-like 4 blockade after transfer into congenic hosts (Fig. 3c). Thus, even though blockade with either anti-jagged 1 or anti delta-like 4 results in increased IL-4 production, the reasons for this increase are different. Anti-jagged 1 treatment causes an increase in memory cell expansion, which produces more IL-4. Anti-delta-like 4 treatment reduces the suppression of effector cell proliferation, which results in a greater number of effector cells and more IL-4 production.

Exposure of dendritic cells to a number of signals can upregulate jagged-1 [34], [35]. It is possible that a downregulation of this molecule at the conclusion of the immune response may help to contract the effector T cell population. Thus Notch ligands could help to regulate the T cell response in several ways and may prove to be of importance in every aspect of T cell biology.

## Supporting Information

Figure S1 Transfer of primary effector (CD44^hi^CD62L^−^) or memory (CD44^hi^CD62L^−^CCR7^+^) cells into a congenic host results in the donor cell expressing cell surface markers demonstrating effector cell phenotype (CD44^hi^CD62L^−^) in both the lung in the lymph node upon in vivo challenge with antigen that both host and donor were sensitized to.
(6.37 MB TIF)

Figure S2 Development of a specific polyclonal antibody to jagged-1. A) To test specificity of this antibody we used both recombinant jagged-1 protein and lysates from OP-9 cells stably transfected with Notch ligands. Shown is the lysate for jagged-1 and jagged-1 as well as recombinant jagged-1. Lysates for cells expressing delta-like 1,3 and 4 were also tested and found to be non-cross reactive. B and C) Anti delta-like 4 is functional in vivo. To determine if our antibody was functional in vivo we analyzed the thymus of mice receiving anti delta-like 4 treatment. Those mice displayed a similar phenotype to that observed when delta-like 4 was depleted from thymic epithelial cells [25]. D) A decrease in IL-10 was observed with anti-jagged 1 blockade during primary immunologic stimulation. This replicates the data published by Elayman et al. [34] and indicates our antibody is blocking efficiently in vivo.
(3.90 MB TIF)

Figure S3 The number of other lymphocyte subsets was not significantly altered in the secondary model of S. Mansoni challenge.
(2.01 MB TIF)

Figure S4 Primary S. Mansoni egg challenge does not have the same effect as egg exposure in previously sensitized mice. A) While there was significant IL-4 production from draining lymph node cells restimulated with SEA (*p = 0.001), there was no significant difference between groups treated with control Ig or anti jagged-1 antibody. B–C) We observed a significant decrease in the number of total and effector CD4^+^ cells in the lymph node, and an increase in the number of total and effector CD4^+^ cells in the lung. *p = 0.05 in all cases. D) Flow plot of lymph node CD45.2^+^ cells demonstrating that they are antigen experienced (CD44^hi^) after transfer into a mouse that was then challenged with OVA. E–F) The number of total CD45.2 cells found in the lung and lymph node after 3 challenges with OVA.
(5.97 MB TIF)

Figure S5 Antigen specific and Th1 cell proliferation is altered with anti jagged-1 treatment. A,B) OTII effector and central memory cells transferred into congenic mice displayed a similar pattern as seen in the secondary S. Mansoni challenge model. *p<0.03 in all cases. C,D) Effector cell proliferation is also altered by anti jagged-1 treatment in a Th1 model of pulmonary inflammation initiated by PPD antigen. *p<0.05.
(6.59 MB TIF)

Figure S6 Delta-like 4 causes down regulation of noxa expression in naive T cells. Cells were stimulated for 4 hours and RNA was analyzed for expression of noxa, a gene suppressed by BMI1. *p = 0.0188
(1.14 MB TIF)
