# Supplementary figures and images for: Delta-Like 4 Differentially Regulates Murine CD4+ T Cell Expansion via BMI1
Source: PLoS One. 2010 Aug 17;5(8):e12172. doi: 10.1371/journal.pone.0012172 (PMC2923143; doi:10.1371/journal.pone.0012172)

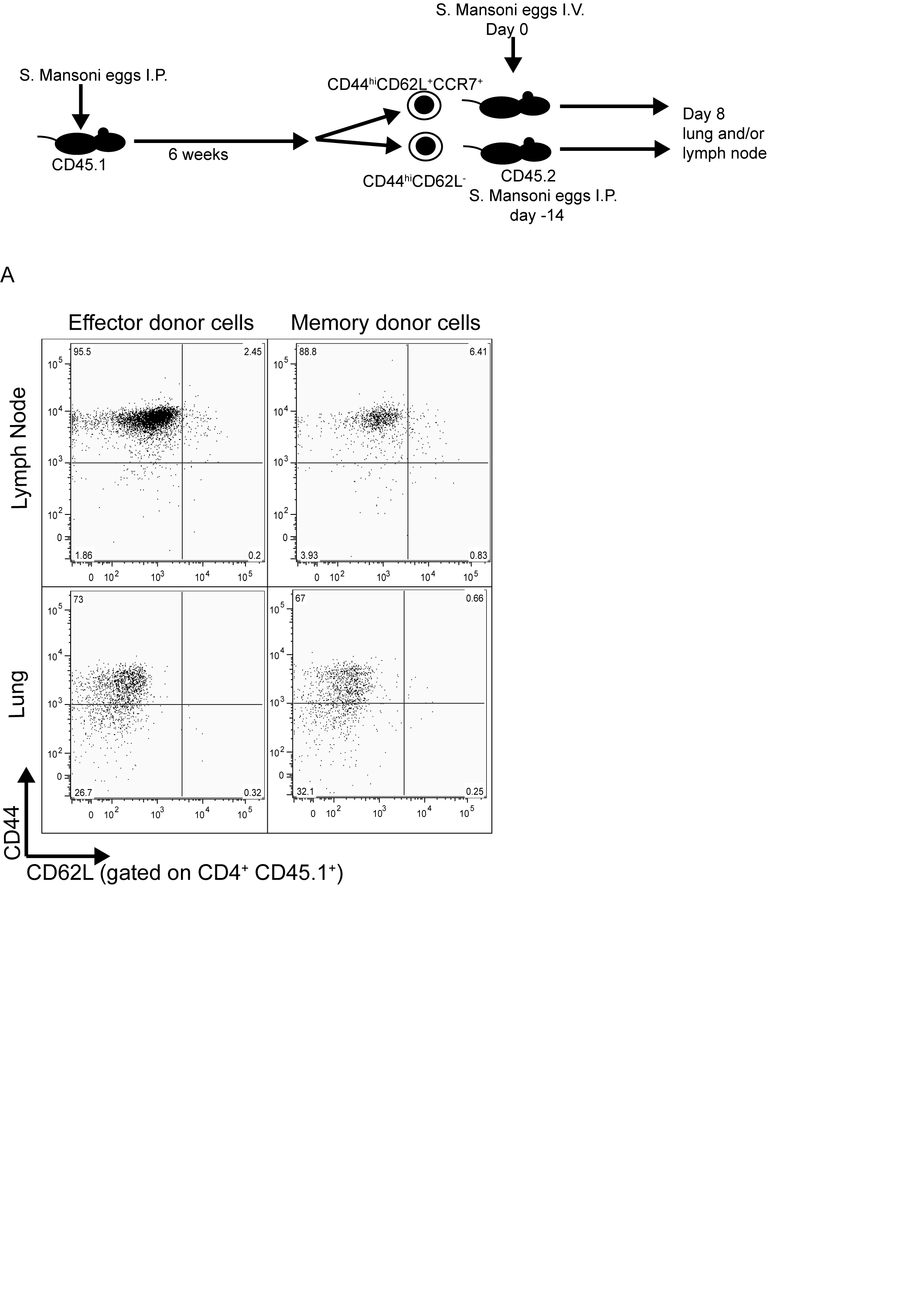

Supplement: Figure S1 — Transfer of primary effector (CD44hiCD62L−) or memory (CD44hiCD62L−CCR7+) cells into a congenic host results in the donor cell expressing cell surface markers demonstrating effector cell phenotype (CD44hiCD62L−) in both the lung in the lymph node upon in vivo challenge with antigen that both host and donor were sensitized to. (6.37 MB TIF) [file pone.0012172.s001.tif]

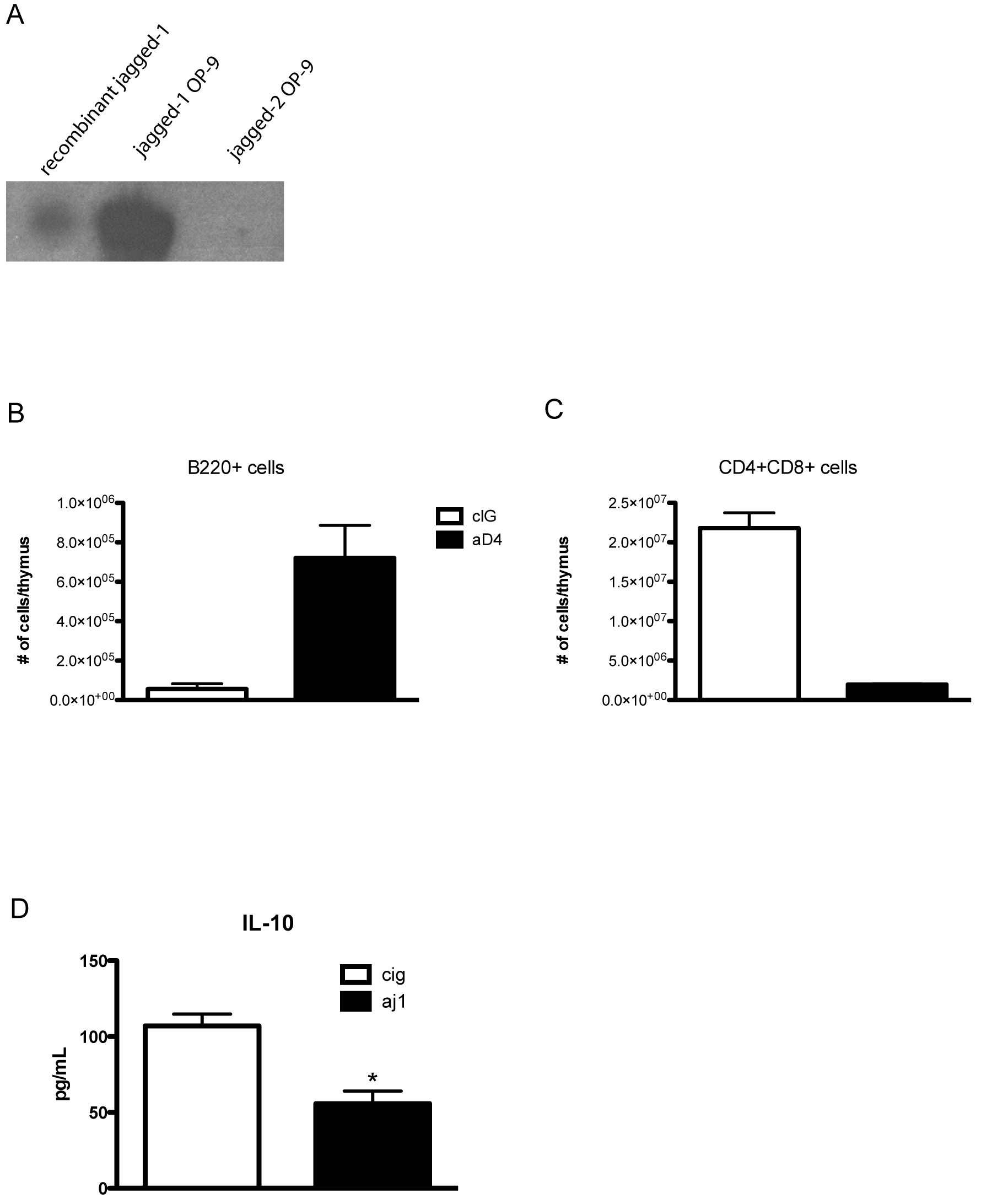

Supplement: Figure S2 — Development of a specific polyclonal antibody to jagged-1. A) To test specificity of this antibody we used both recombinant jagged-1 protein and lysates from OP-9 cells stably transfected with Notch ligands. Shown is the lysate for jagged-1 and jagged-1 as well as recombinant jagged-1. Lysates for cells expressing delta-like 1,3 and 4 were also tested and found to be non-cross reactive. B and C) Anti delta-like 4 is functional in vivo. To determine if our antibody was functional in vivo we analyzed the thymus of mice receiving anti delta-like 4 treatment. Those mice displayed a similar phenotype to that observed when delta-like 4 was depleted from thymic epithelial cells [25]. D) A decrease in IL-10 was observed with anti-jagged 1 blockade during primary immunologic stimulation. This replicates the data published by Elayman et al. [34] and indicates our antibody is blocking efficiently in vivo. (3.90 MB TIF) [file pone.0012172.s002.tif]

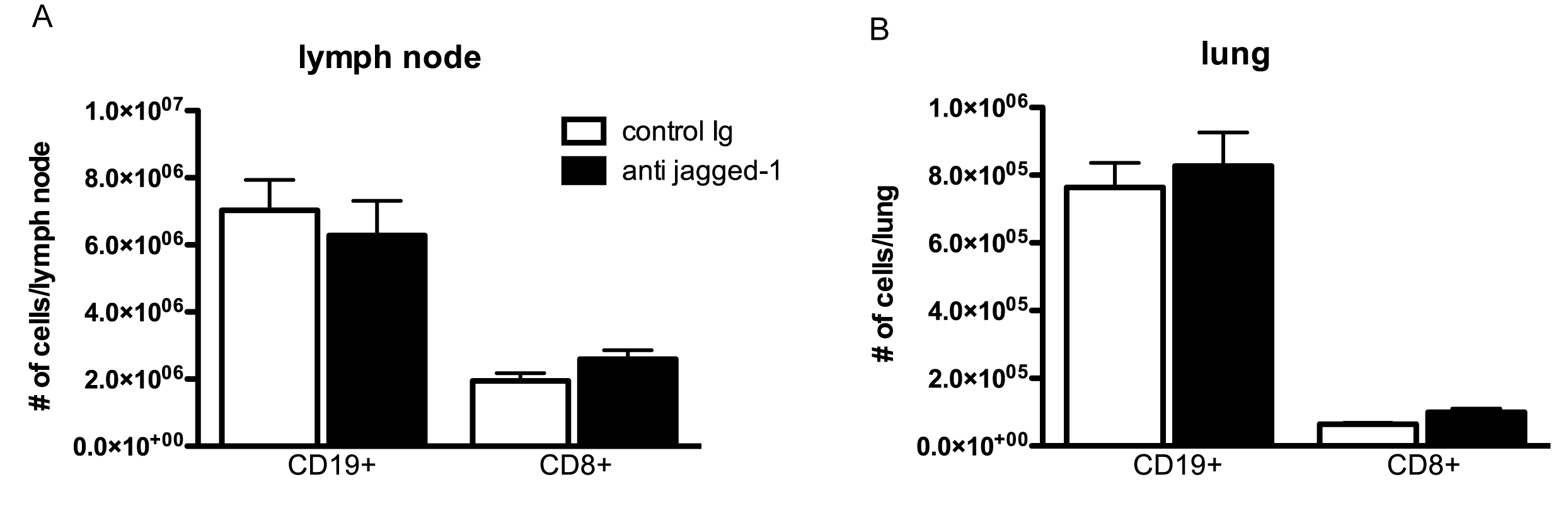

Supplement: Figure S3 — The number of other lymphocyte subsets was not significantly altered in the secondary model of S. Mansoni challenge. (2.01 MB TIF) [file pone.0012172.s003.tif]

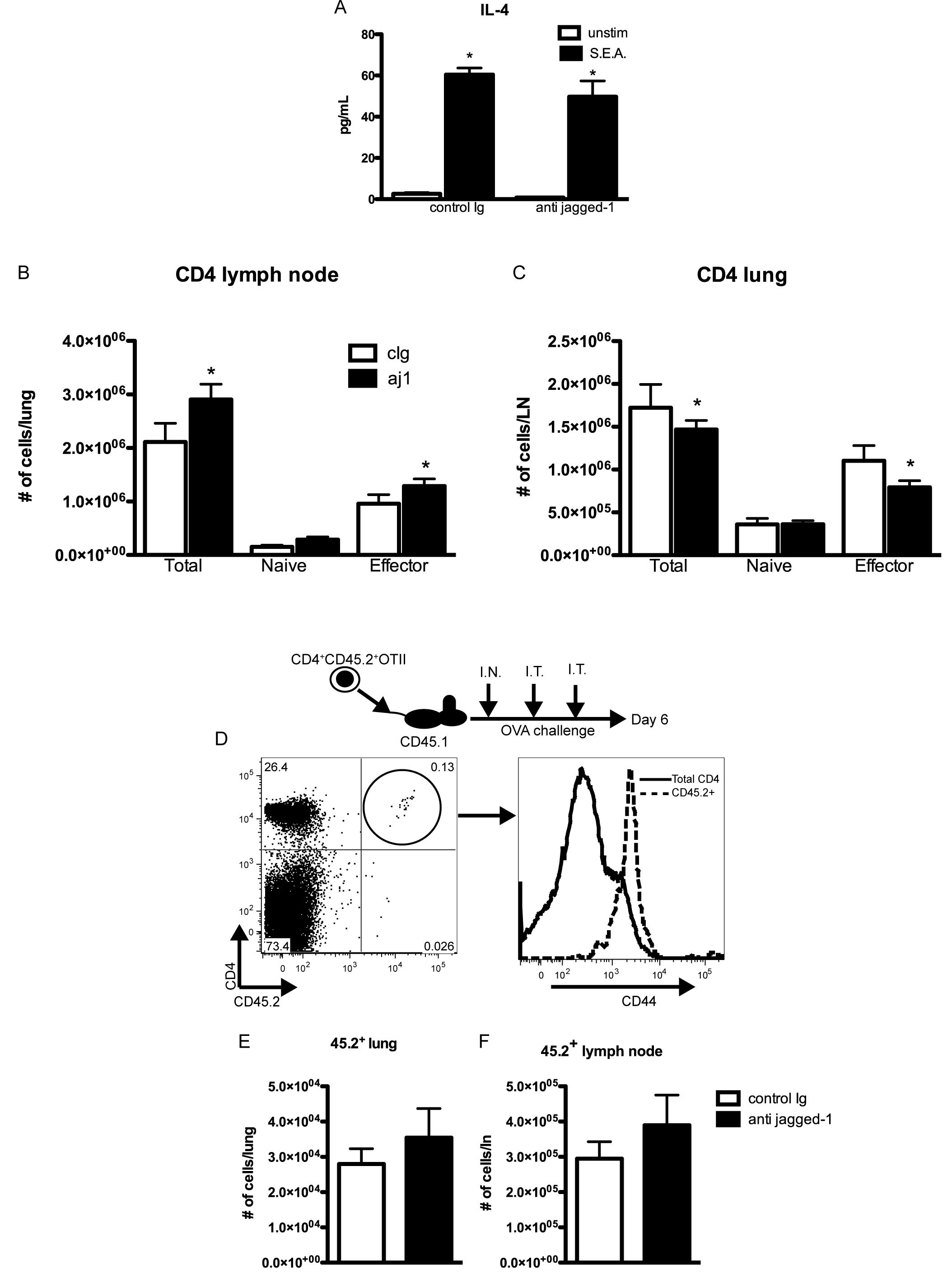

Supplement: Figure S4 — Primary S. Mansoni egg challenge does not have the same effect as egg exposure in previously sensitized mice. A) While there was significant IL-4 production from draining lymph node cells restimulated with SEA (*p = 0.001), there was no significant difference between groups treated with control Ig or anti jagged-1 antibody. B–C) We observed a significant decrease in the number of total and effector CD4+ cells in the lymph node, and an increase in the number of total and effector CD4+ cells in the lung. *p = 0.05 in all cases. D) Flow plot of lymph node CD45.2+ cells demonstrating that they are antigen experienced (CD44hi) after transfer into a mouse that was then challenged with OVA. E–F) The number of total CD45.2 cells found in the lung and lymph node after 3 challenges with OVA. (5.97 MB TIF) [file pone.0012172.s004.tif]

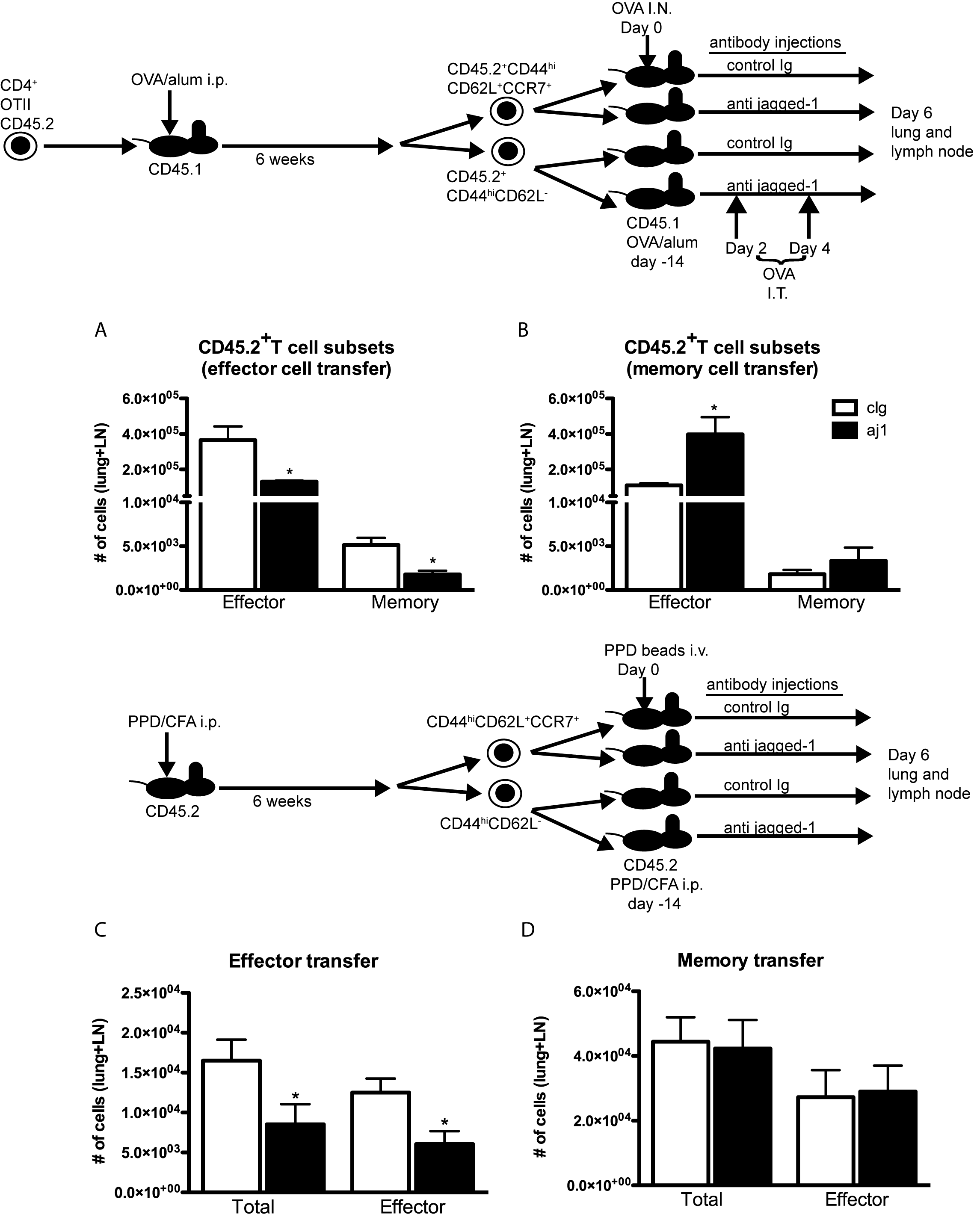

Supplement: Figure S5 — Antigen specific and Th1 cell proliferation is altered with anti jagged-1 treatment. A,B) OTII effector and central memory cells transferred into congenic mice displayed a similar pattern as seen in the secondary S. Mansoni challenge model. *p<0.03 in all cases. C,D) Effector cell proliferation is also altered by anti jagged-1 treatment in a Th1 model of pulmonary inflammation initiated by PPD antigen. *p<0.05. (6.59 MB TIF) [file pone.0012172.s005.tif]

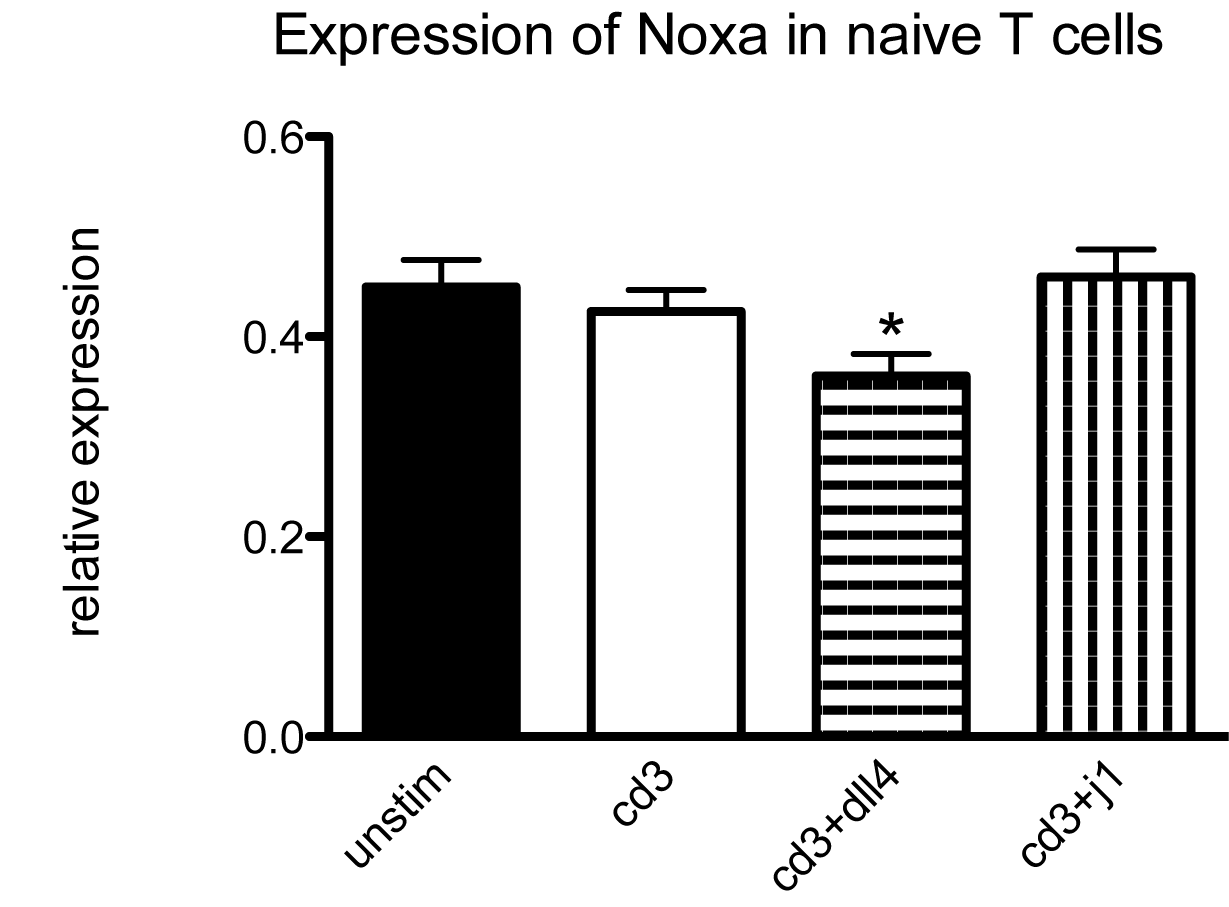

Supplement: Figure S6 — Delta-like 4 causes down regulation of noxa expression in naive T cells. Cells were stimulated for 4 hours and RNA was analyzed for expression of noxa, a gene suppressed by BMI1. *p = 0.0188 (1.14 MB TIF) [file pone.0012172.s006.tif]
